# Postprandial 2-h glucose tolerance is associated with diabetes diagnosis, diabetes mortality, and cardiovascular mortality

**DOI:** 10.1038/s41598-025-28849-y

**Published:** 2025-12-15

**Authors:** Yutang Wang, Yan Fang, Guang Yang, Francesco Prattichizzo, Antonio Ceriello

**Affiliations:** 1 Discipline of Life Science, Institute of Innovation, Science and Sustainability, Federation University Australia, University Drive, Mt Helen, Ballarat, VIC 3350 Australia; 2 Department of Gerontology, The First Affiliated Hospital, Shandong First Medical University, Jinan, 250014 Shandong China; 3 IRCCS MultiMedica, Milan, Italy

**Keywords:** Postprandial, Oral glucose tolerance test, Cardiovascular disease, Diabetes, Survival, Biomarkers, Cardiology, Endocrinology, Risk factors, Cardiovascular diseases, Metabolic disorders

## Abstract

Postprandial plasma glucose between 4 and 7.9 h is associated with the diagnosis of diabetes, diabetes mortality, and cardiovascular mortality. However, it is unknown whether 2-hour plasma glucose during the oral glucose tolerance test conducted in this postprandial period (4–7.9 h), termed as 2-h PG_OGTT@4–7.9 h_, can accurately classify diabetes diagnosis and predict mortality risks. This study aimed to address these questions using 2,347 adult participants. Diabetes was defined as HbA_1c_ ≥ 6.5%, and the ability of 2-h PG_OGTT@4–7.9 h_ to classify diabetes was analyzed using receiver operating characteristic curves. Cox proportional hazards models were employed to estimate mortality hazard ratios (HRs) and 95% confidence intervals (CIs). The results showed that 2-h PG_OGTT@4–7.9 h_ could classify diabetes with 92% accuracy. Participants were followed up for a mean of 21.4 years. A 1-square-root higher 2-h PG_OGTT@4–7.9 h_ was associated with an increased risk of mortality from all causes (adjusted HR, 1.06; 95% CI, 1.04–1.08), diabetes (adjusted HR, 1.46; 95% CI, 1.33–1.61), and cardiovascular disease (adjusted HR, 1.07; 95% CI, 1.03–1.11). In conclusion, 2-h PG_OGTT@4–7.9 h_, a non-fasting test, may be useful for diabetes classification and prediction of mortality risk from diabetes and cardiovascular disease.

**Supplementary Information:**

The online version contains supplementary material available at 10.1038/s41598-025-28849-y.

## Introduction

Diabetes is a metabolic disease characterized by elevated blood glucose levels^1^. As of 2021, approximately 529 million people worldwide were living with diabetes, a number projected to rise to 1.31 billion by 2050^2^. This condition contributes to about 1.5 million deaths annually^3^. It imposes a substantial economic burden, costing $1.3 trillion globally in 2015, which figure is estimated to climb to around $2.2 trillion by 2030^4^.

In 2021, about half of diabetic cases in adults remained undiagnosed^5^. Those with undiagnosed diabetes are developing diabetes-related complications, leading to increased healthcare expenditure^6^. Individuals with undiagnosed diabetes face a 60% higher risk of mortality compared to those without diabetes^7^. Timely diagnosis is crucial for initiating appropriate medical interventions to prevent or delay diabetes-related complications^8^. Therefore, enhanced efforts are needed to improve diabetes detection.

Currently, diabetes diagnosis relies on fasting plasma glucose levels, 2-h plasma glucose during an oral glucose tolerance test (OGTT), and hemoglobin A_1c_ (HbA_1c_)^9^. However, fasting requirements for tests such as fasting plasma glucose and OGTT can be inconvenient and may induce hypoglycemia in vulnerable individuals^10^. Exploring the diagnostic potential of non-fasting plasma glucose and non-fasting OGTT could therefore offer valuable insights.

Recent research highlights postprandial glucose levels measured between 4 and 7.9 h after a meal (PPG_4–7.9 h_) as a promising biomarker for diagnosis. Computed PPG_4–7.9 h_ demonstrates an 87% accuracy in diagnosing diabetes^11^, falling within the optimal accuracy range of 80% to 90%^12^. Moreover, PPG_4–7.9 h_ has been linked to predicting mortality from diabetes, cardiovascular disease (CVD)^13^, and cancer^14^. Importantly, it remains stable throughout this postprandial period, as evidenced by consistent hourly measurements^13,15^.

Supporting this finding, Eichenlau et al.‘s study showed that plasma glucose returned to baseline levels within 4 h after a meal, regardless of meal type (standard meal or high carbohydrate meal) and meal time (breakfast, lunch or dinner) in healthy individuals^16^. These clinical results underscore the potential of the postprandial period between 4 and 7.9 h to reflect an individual’s glucose homeostasis state, offering a promising window for diabetes diagnosis.

Yet, the diagnostic and prognostic value of 2-h plasma glucose during OGTT conducted within this postprandial period between 4 and 7.9 h (2-h PG_OGTT@4–7.9 h_) remains unknown. This study aimed to explore whether 2-h PG_OGTT@4–7.9 h_ was associated with diabetes diagnosis and predicted mortality risks. It utilized data from 2,347 adult participants who attended the third National Health and Nutrition Examination Survey (NHANES III) during 1988–1994. Additionally, 3,865 participants from the same survey with 2-h plasma glucose during OGTT conducted in the fasting period (fasting time ≥ 8 h^9,17,18^), termed as 2-h PG_OGTT@fasting_, were included in the analysis.

## Methods

### Participants

This study included adult participants (aged ≥ 20 years) from NHANES III (1988–1994)^19^. Two cohorts of participants were selected from the participants: the postprandial cohort (fasting time, 4–7.9 h) and the fasting cohort (fasting time, ≥ 8 h^9,17,18^).

**Fig. 1:**
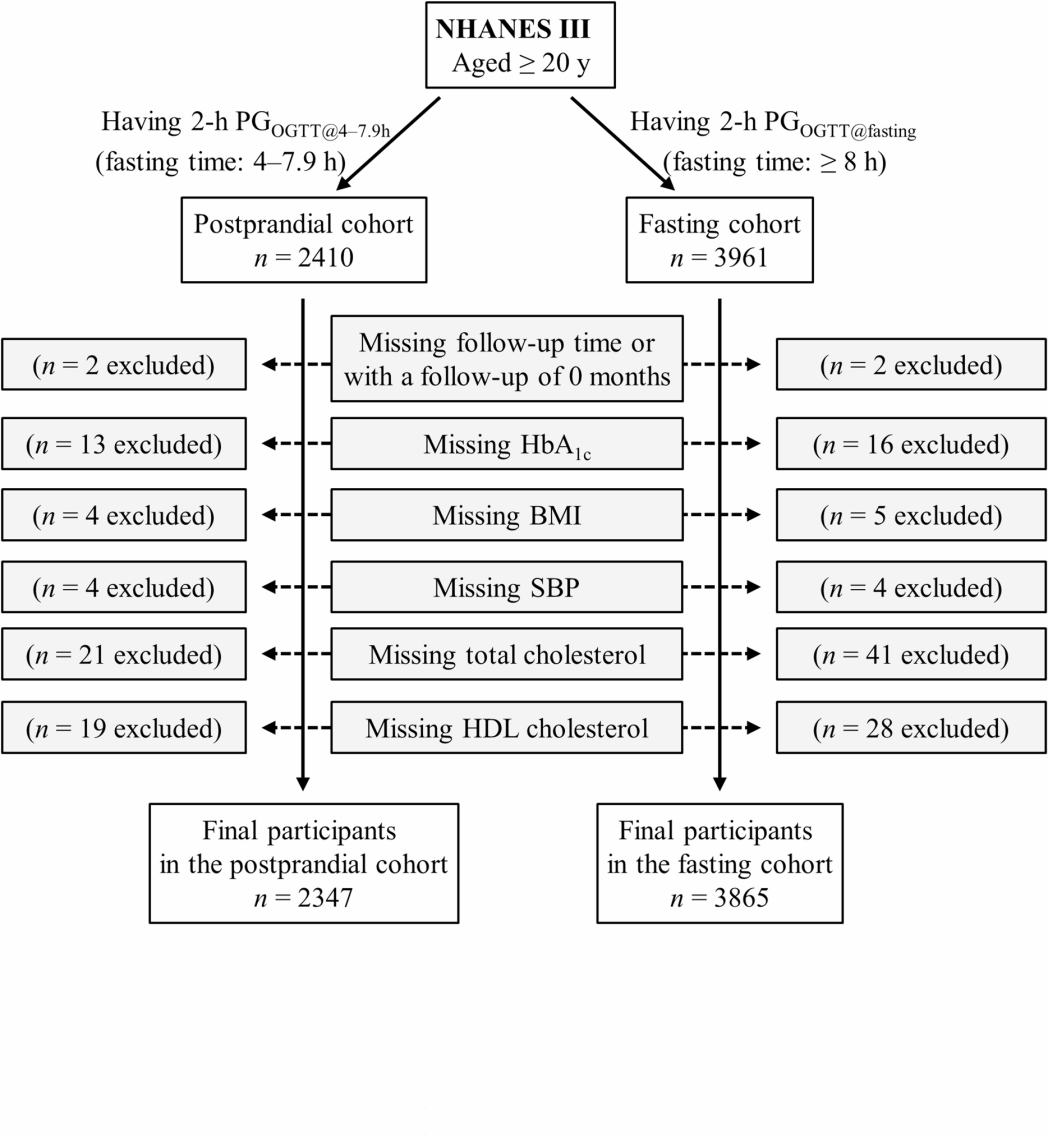
Flow diagram of the study participants. 2-h PG_OGTT@4–7.9 h_, 2-h plasma glucose during OGTT conducted in the postprandial period between 4 and 7.9 h; 2-h PG_OGTT@fasting_, 2-h plasma glucose during OGTT conducted in the fasting period (fasting time, ≥ 8 h); BMI, body mass index; HbA_1c_, hemoglobin A_1c_; HDL, high-density lipoprotein; NHANES III, the third National Health and Nutrition Examination Survey; OGTT, oral glucose tolerance test; PG, plasma glucose; SBP, systolic blood pressure.

The postprandial cohort included all participants who had 2-h plasma glucose during OGTT conducted in the postprandial period between 4 and 7.9 h (*n* = 2410). This 2-h plasma glucose was termed as 2-h PG_OGTT@4–7.9 h_. Participants missing follow-up time or with a follow-up of 0 months (*n =* 2) were excluded. Individuals who lacked the following data were also excluded: HbA_1c_ (*n =* 13), body mass index (*n =* 4), systolic blood pressure (*n =* 4), total cholesterol (*n =* 21), and high-density lipoprotein (HDL) cholesterol (*n =* 19). Therefore, the remaining 2347 participants were included in the final analysis for the postprandial cohort (Fig. 1).

The fasting cohort included all participants who had 2-h plasma glucose during OGTT conducted in the fasting period (fasting time, ≥ 8 h; *n* = 3961). The 2-h plasma glucose in this cohort was termed as 2-h PG_OGTT@fasting_. Participants missing follow-up time or with a follow-up of 0 months (*n =* 2) were excluded. Individuals who lacked the following data were also excluded: HbA_1c_ (*n =* 16), body mass index (*n =* 5), systolic blood pressure (*n =* 4), total cholesterol (*n =* 41), and HDL cholesterol (*n =* 28). Therefore, the remaining 3865 participants were included in the final analysis for the fasting cohort (Fig. 1).

### Ethical considerations

The study was conducted in accordance with the Declaration of Helsinki and approved by the NHANES Institutional Review Board. All participants provided written informed consent. All participant records were anonymised prior to being accessed by the authors.

### Exposure variable

The exposure variable of this study was 2-h plasma glucose during OGTT which was conducted in the postprandial period between 4 and 7.9 h or in the fasting period (fasting time, ≥ 8 h^9,17,18^). During the OGTT test, participants were administered a glucose challenge containing the equivalent of 75 g of glucose^20^. Two hours later, a blood specimen was drawn to measure 2-h plasma glucose levels using the hexokinase method^21,22^.

### Outcome variables

The outcome variables of this study were HbA_1c_, diabetes diagnosis, and various types of mortality.

HbA_1c_ was measured using the Bio-Rad DIAMAT glycosylated hemoglobin analyzer system^21^. Currently, diabetes in the clinic is diagnosed using HbA_1c_, fasting plasma glucose and 2-h plasma glucose during OGTT which was conducted in the fasting period. However, participants in the postprandial cohort lacked fasting plasma glucose and OGTT that was conducted in the fasting period. Therefore, diabetes in the current study was defined as HbA_1c_ ≥ 6.5 only in the main analyses. Diabetes was also defined as a self-reported diagnosis in additional analyses.

Data on mortality from diabetes, CVD, cancer, and all causes were directly retrieved from NHANES-linked mortality files^19^. To evaluate mortality status and the cause of death, the National Center for Health Statistics linked the NHANES data with death certificate records from the National Death Index records^23^. Follow-up time was the duration from the time when the individual was examined at the Mobile Examination Center until death or until the conclusion of follow-up (31 December 2019), whichever occurred first^24^.

### Covariables

Covariables were described previously^11,15^ and included age, sex (female or male), ethnicity (non-Hispanic white, non-Hispanic black, Hispanic, or other), body mass index, poverty–income ratio (< 130%, 130%–349%, ≥ 350%, or unknown), education (< high school, high school, > high school, or unknown), smoking status (current smoker, past smoker, or non-smoker), alcohol consumption (never, < 1 drink per week, 1–6 drinks per week, ≥ 7 drinks per week, or unknown), physical activity (inactive, insufficiently active, or active), survey periods (1988–1991 or 1991–1994), systolic blood pressure, total cholesterol, high-density lipoprotein (HDL) cholesterol, and family history of diabetes (yes, no, or unknown).

### Statistical analyses

The baseline characteristics of these two cohorts of participants were presented as median and interquartile range for not normally distributed continuous variables, mean and standard deviation for normally distributed continuous variables, or number and percentage for categorical variables^25^. Differences in continuous variables were analyzed via the Mann-Whitney U test (not normally distributed)^26^ and Student’s T-test (normally distributed)^27^, and differences among categorical variables were analyzed via Pearson’s chi-square test^28^.

The associations of 2-h plasma glucose with HbA_1c_ and diabetes diagnosis were analyzed by multiple linear regression and binary logistic regression^29,30^, respectively. Receiver operating characteristic curves were constructed and the area under the curve (AUC) was calculated to assess the association of 2-h plasma glucose with diabetes diagnosis and mortality^31^, and the Youden Index was used to determine the optimal cutoff^32^.

Cox proportional hazards models were used to calculate hazard ratios (HRs) and 95% confidence intervals (CIs) of 2-h plasma glucose for mortality from diabetes, CVD, cancer, and all causes. 2-h plasma glucose was treated as a continuous variable (natural log-transformed) or categorical variable (≥ versus < 200 mg/dL, or < 140 versus ≥ 140 & <200 mg/dL). Kaplan–Meier curves were constructed to estimate the survival rates of participants between the two 2-h plasma glucose categories (≥ versus < 200 mg/dL), which were compared using the log-rank test^33^. To improve data distribution, body mass index, total cholesterol, HDL cholesterol, and systolic blood pressure were natural log-transformed and 2-h plasma glucose was square root-transformed in all the regression analyses^34^.

Power estimation was conducted by simulations employing 10,000 randomly generated samples with various sample sizes (ranging from 50 to 200) derived from the postprandial cohort of 2347 participants^35,36^. Diabetes prediction was defined as a 2-h PG_OGTT@4–7.9 h_ ≥ 200 mg/dL, and actual diabetes status was defined as HbA_1c_ ≥ 6.5%^37^. Within each sample, the diagnostic accuracy, sensitivity, and specificity of 2-h PG_OGTT@4–7.9 h_ for diabetes diagnosis were then calculated^38–40^.

A diagnostic accuracy of 80%, which is deemed a minimum threshold for an excellent diagnostic marker^12^, was used for power estimation. The percentage of samples exhibiting ≥ 80% accuracy out of 10,000 random samples was assigned as the diagnostic power of 2-h PG_OGTT@4–7.9 h_ in classifying diabetes. Mean sensitivity and specificity values were calculated from the 10,000 samples, and their 95% confidence intervals were generated from the 2.5th and 97.5th percentiles of the 10,000 sensitivity and specificity values^41^. In addition, a diagnostic accuracy of 81% was also used to estimate power and sample size.

The null hypothesis was rejected for two-sided values of *p* < 0.05. The estimation of power and sample size were conducted using the R program, and the remaining analyses were conducted using SPSS version 27.0 (IBM SPSS Statistics for Windows, Armonk, NY, USA, IBM Corporation)^42^.

## Results

### Baseline characteristics

This study included two cohorts of participants: the postprandial cohort (fasting time, 4–7.9 h; *n* = 2347) and the fasting cohort (fasting time, ≥ 8 h; *n* = 3865). Both cohorts had a mean age of 56 years. Participants with higher 2-h plasma glucose during OGTT were older, and had higher levels of HbA_1c_, body mass index, systolic blood pressure, and total cholesterol, and had lower levels of HDL-cholesterol, education, and income (Supplementary Tables S1-S2 online).

### Association of 2-h plasma glucose during OGTT with HbA_1c_

2-h PG_OGTT@4–7.9 h_ was positively associated with HbA_1c_ without adjustment (Model 1, β = 0.614, *p* < 0.001, Supplementary Table S3 online). This association remained significant after adjustment for all the tested confounders (Model 6, β = 0.606, *p* < 0.001, Supplementary Table S3). Similarly, 2-h PG_OGTT@fasting_ was positively associated with HbA_1c_ in the absence (β = 0.671) and presence of adjustment (β = 0.650, Supplementary Table S3 online).

### Association of 2-h plasma glucose during OGTT with diabetes diagnosis

A 1-square-root increase in 2-h PG_OGTT@4–7.9 h_ was associated with a higher risk of HbA_1c_-diagnosed diabetes after adjustment for all the tested confounders (Model 6; OR = 2.44; 95% CI, 2.19–2.73; *p* < 0.001; Table 1). 2-h PG_OGTT@fasting_ was associated with HbA_1c_-diagnosed diabetes to a similar extent (Model 6; OR = 2.49; 95% CI, 2.28–2.71; *p* < 0.001; Table 1).

**Table 1:** Association of 2-h plasma glucose during OGTT (square root-transformed) with diabetes diagnosis (defined as HbA_1c_ ≥ 6.5%).

Models	2-h PG_OGTT@4–7.9 h_			2-h PG_OGTT@fasting_		
	OR	95% CI	*p*	OR	95% CI	*p*
Model 1	2.29	2.09–2.50	< 0.001	2.31	2.15–2.48	< 0.001
Model 2	2.38	2.16–2.63	< 0.001	2.47	2.28–2.68	< 0.001
Model 3	2.36	2.14–2.61	< 0.001	2.46	2.26–2.66	< 0.001
Model 4	2.44	2.20–2.71	< 0.001	2.51	2.31–2.74	< 0.001
Model 5	2.44	2.19–2.72	< 0.001	2.49	2.28–2.71	< 0.001
Model 6	2.44	2.19–2.73	< 0.001	2.49	2.28–2.71	< 0.001

Model 1 was not adjusted; Model 2 was adjusted for age, sex, and ethnicity; Model 3 was adjusted for all the factors in Model 2 plus body mass index, poverty–income ratio, and education; Model 4 was adjusted for all the factors in Model 3 plus physical activity, alcohol consumption, smoking status, and survey period; Model 5 was adjusted for all the factors in Model 4 plus total cholesterol, HDL cholesterol, and systolic blood pressure; and Model 6 was adjusted for all the factors in Model 5 plus family history of diabetes.

2-h PG_OGTT@4–7.9 h_, 2-hour plasma glucose during OGTT which was conducted in the postprandial period between 4 and 7.9 h; 2-h PG_OGTT@fasting_, 2-hour plasma glucose during OGTT which was conducted in the fasting period (fasting time, ≥ 8 h); CI, confidence interval; HbA_1c_, hemoglobin A_1c_; OGTT, oral glucose tolerance test; OR, odds ratio.

**Fig. 2:**
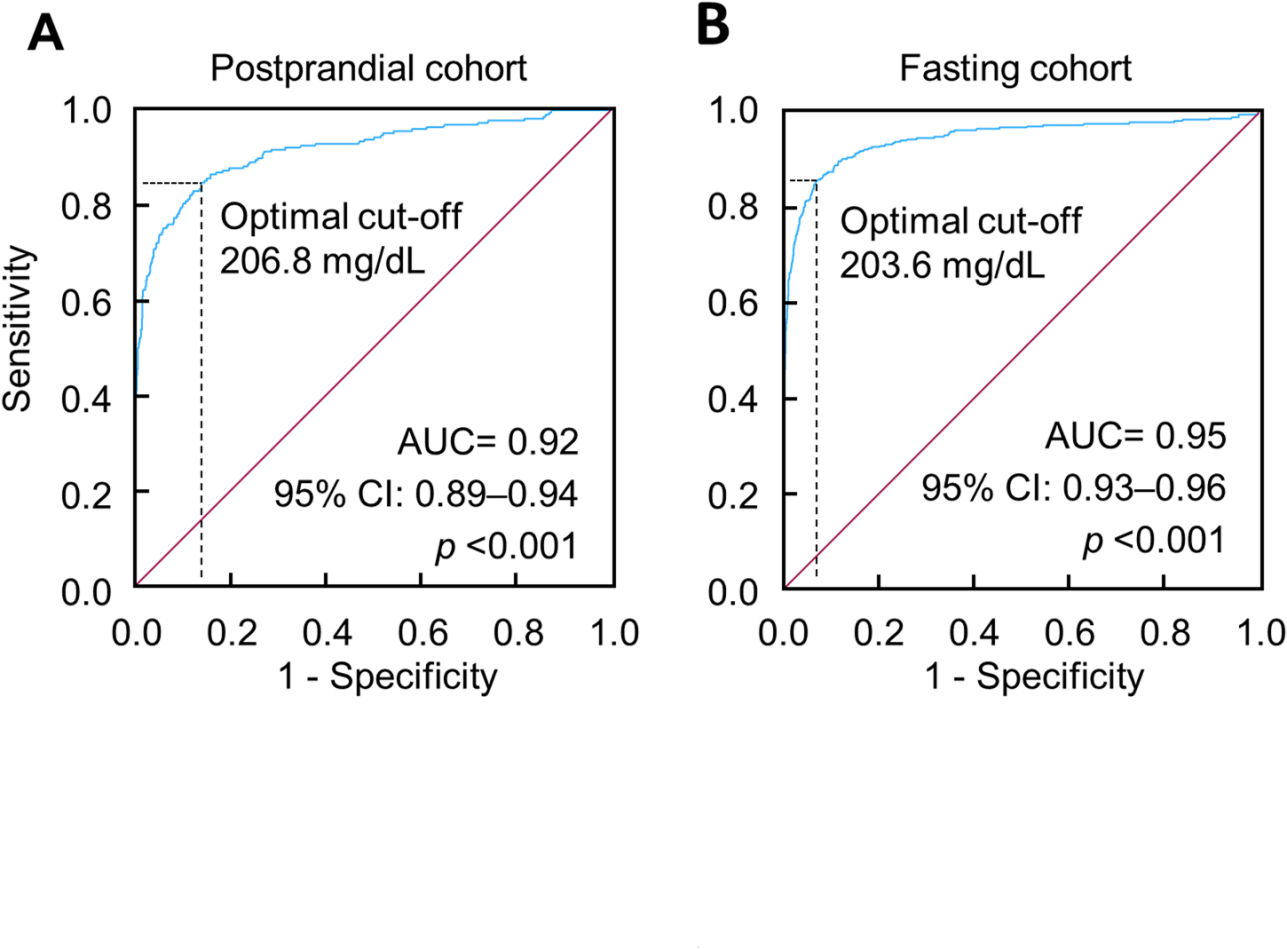
ROC curves of 2-h plasma glucose to classify diabetes, defined as HbA_1c_ ≥ 6.5%. (**A**) OGTT was conducted in the postprandial period between 4 and 7.9 h. The optimal cutoff was 206.8 mg/dL, with a sensitivity of 84.8%, specificity of 86.1%, and an area under the curve (AUC) of 0.92. (**B**) OGTT was conducted in the fasting period (fasting time, ≥ 8 h). The optimal cutoff was 203.6 mg/dL, with a sensitivity of 85.8%, specificity of 93.1%, and an AUC of 0.95. HbA_1c_, hemoglobin A_1c_; OGTT, oral glucose tolerance test; ROC, receiver operating characteristic.

ROC curve analysis showed that 2-h PG_OGTT@4–7.9 h_ predicted HbA_1c_-diagnosed diabetes with an accuracy of 92% as indicated by the AUC value, and the accuracy for 2-h PG_OGTT@fasting_ was 95% (Fig. 2). The optimal cutoff for 2-h PG_OGTT@4–7.9 h_ to predict HbA_1c_-diagnosed diabetes was 206.8 mg/dL, and the corresponding cutoff for 2-h PG_OGTT@fasting_ was 203.6 mg/dL (Fig. 2).

In further analyses, we defined diabetes as a self-reported diagnosis. The results showed that both 2-h PG_OGTT@4–7.9 h_ and 2-h PG_OGTT@fasting_ remained significantly associated with diabetes diagnosis (Supplementary Table S4 and Supplementary Figure S1).

### Association of 2-h plasma glucose during OGTT with Pre-diabetes diagnosis

Prediabetes was defined as HbA_1c_ ranging between 5.7% and 6.4%. A 1-square-root increase in 2-h PG_OGTT@4–7.9 h_ was associated with a 13% increased risk of pre-diabetes diagnosis after adjustment for all the tested confounders (Model 6; OR, 1.13.1; 95% CI, 1.07–1.19; *p* < 0.001; Supplementary Table S5 online). When 2-h plasma glucose during OGTT was treated as a dichotomous variable using the cut off of 140 mg/dL, those with 2-h PG_OGTT@4–7.9 h_ ≥140 mg/dL had a 29% higher risk of pre-diabetes diagnosis after adjustment for all the tested confounders (Model 6; OR, 1.29.1; 95% CI, 1.04–1.60; *p* = 0.021; Table 2). As expected, 2-h PG_OGTT@fasting_ was associated with a higher risk of HbA_1c_-defined pre-diabetes.

**Table 2:** Association of 2-h plasma glucose during OGTT (categorical, < 140 versus ≥ 140 mg/dL) with pre-diabetes diagnosis.

Models	2-h PG_OGTT@4–7.9 h_			2-h PG_OGTT@fasting_		
	OR	95% CI	*p*	OR	95% CI	*p*
Model 1	1.42	1.18–1.71	< 0.001	2.22	1.91–2.58	< 0.001
Model 2	1.39	1.13–1.70	0.002	2.03	1.73–2.38	< 0.001
Model 3	1.29	1.04–1.59	0.018	1.90	1.61–2.24	< 0.001
Model 4	1.36	1.10–1.69	0.004	2.00	1.69–2.36	< 0.001
Model 5	1.30	1.04–1.61	0.019	2.01	1.70–2.39	< 0.001
Model 6	1.29	1.04–1.60	0.021	2.00	1.68–2.37	< 0.001

Pre-diabetes diagnosis was defined as HbA_1c_ ranging between 5.7% and 6.4%. Out of 2347 participants in the postprandial cohort, 230 had an HbA_1c_ value of ≥ 6.5% and were excluded from the analysis. So, a total of 2117 participants were included in the final analysis. 2-h PG_OGTT@4–7.9 h_, 2-hour plasma glucose during OGTT which was conducted in the postprandial period between 4 and 7.9 h; 2-h PG_OGTT@fasting_, 2-hour plasma glucose during OGTT which was conducted in the fasting period (fasting time, ≥ 8 h); CI, confidence interval; HbA_1c_, hemoglobin A_1c_; OGTT, oral glucose tolerance test; OR, odds ratio.

Model 1 was not adjusted; Model 2 was adjusted for age, sex, and ethnicity; Model 3 was adjusted for all the factors in Model 2 plus body mass index, poverty–income ratio, and education; Model 4 was adjusted for all the factors in Model 3 plus physical activity, alcohol consumption, smoking status, and survey period; Model 5 was adjusted for all the factors in Model 4 plus total cholesterol, HDL cholesterol, and systolic blood pressure; and Model 6 was adjusted for all the factors in Model 5 plus family history of diabetes.

### Association of 2-h plasma glucose during OGTT with diabetes mortality

The postprandial cohort was followed up for 50,185 person-years with a mean follow-up of 21.4 years. The fasting cohort was followed up for 82,039 person-years with a mean follow-up of 21.2 years. During the follow-up, diabetes led to 40 and 62 deaths in the postprandial and the fasting cohorts, respectively (Supplementary Table S6).

A 1-square-root increase in 2-h PG_OGTT@4–7.9 h_ was associated with a 46% increase in diabetes mortality risk after adjustment for all the tested confounders (Model 6; HR, 1.46.1; 95% CI, 1.33–1.61; *p* < 0.001; Table 3). A 1-square-root increase in 2-h PG_OGTT@fasting_ was associated with a 29% increase in diabetes mortality risk after adjustment for all the tested confounders (Model 6; HR, 1.29; 95% CI, 1.21–1.38; *p* < 0.001; Table 3).

**Table 3:** Association of 2-h plasma glucose (square root-transformed) with diabetes mortality.

Models	2-h PG_OGTT@4–7.9 h_			2-h PG_OGTT@fasting_		
	HR	95% CI	*p*	HR	95% CI	*p*
Model 1	1.41	1.32–1.51	< 0.001	1.35	1.28–1.43	< 0.001
Model 2	1.40	1.30–1.52	< 0.001	1.32	1.25–1.40	< 0.001
Model 3	1.40	1.29–1.52	< 0.001	1.31	1.23–1.40	< 0.001
Model 4	1.46	1.34–1.60	< 0.001	1.31	1.23–1.40	< 0.001
Model 5	1.47	1.33–1.62	< 0.001	1.30	1.21–1.38	< 0.001
Model 6	1.46	1.33–1.61	< 0.001	1.29	1.21–1.38	< 0.001

2-h PG_OGTT@4–7.9 h_, 2-hour plasma glucose during OGTT which was conducted in the postprandial period between 4 and 7.9 h; 2-h PG_OGTT@fasting_, 2-hour plasma glucose during OGTT which was conducted in the fasting period (fasting time, ≥ 8 h); CI, confidence interval; HR, hazard ratio; OGTT, oral glucose tolerance test.

Model 1 was not adjusted; Model 2 was adjusted for age, sex, and ethnicity; Model 3 was adjusted for all the factors in Model 2 plus body mass index, poverty–income ratio, and education; Model 4 was adjusted for all the factors in Model 3 plus physical activity, alcohol consumption, smoking status, and survey period; Model 5 was adjusted for all the factors in Model 4 plus total cholesterol, HDL cholesterol, and systolic blood pressure; and Model 6 was adjusted for all the factors in Model 5 plus family history of diabetes.

Further analysis was conducted by treating 2-h plasma glucose as a categorical variable using the clinical cutoff of 200 mg/dL. The Kaplan–Meier survival curves showed that those with 2-h plasma glucose of ≥ 200 mg/dL (versus < 200 mg/dL) had an increased risk of diabetes mortality in both cohorts (*p* < 0.001, Supplementary Figure S2). The positive association remained after further adjustment for all the tested confounders (Supplementary Table S7).

### Association of 2-h plasma glucose during OGTT with All-Cause mortality, CVD mortality, and cancer mortality

We further analyzed the association of 2-h PG_OGTT@4–7.9 h_ with mortality from all causes and CVD. The results showed that a 1-square-root increase in 2-h PG_OGTT@4–7.9 h_ was associated with a 6% increase in multivariable-adjusted risk of all-cause mortality (Model 6; HR, 1.06; 95% CI, 1.04–1.08; *p* < 0.001; Table 4) and a 7% increase in multivariable-adjusted risk of CVD mortality (Model 6; HR, 1.07; 95% CI, 1.03–1.11; *p* < 0.001; Table 5). 2-h PG_OGTT@fasting_ predicted mortality from all causes and CVD to a similar extent (Tables 4 and 5). In addition, neither 2-h PG_OGTT@4–7.9 h_ nor 2-h PG_OGTT@fasting_ was independently associated with cancer mortality (Supplementary Table S8).

**Table 4:** Association of 2-h plasma glucose during OGTT (square root-transformed) with all-cause mortality.

Models	2-h PG_OGTT@4–7.9 h_			2-h PG_OGTT@fasting_		
	HR	95% CI	*p*	HR	95% CI	*p*
Model 1	1.10	1.08–1.12	< 0.001	1.11	1.09–1.12	< 0.001
Model 2	1.06	1.04–1.08	< 0.001	1.06	1.05–1.08	< 0.001
Model 3	1.05	1.03–1.08	< 0.001	1.06	1.04–1.08	< 0.001
Model 4	1.07	1.05–1.09	< 0.001	1.07	1.05–1.08	< 0.001
Model 5	1.06	1.04–1.08	< 0.001	1.06	1.04–1.07	< 0.001
Model 6	1.06	1.04–1.08	< 0.001	1.06	1.04–1.07	< 0.001

2-h PG_OGTT@4–7.9 h_, 2-hour plasma glucose during OGTT which was conducted in the postprandial period between 4 and 7.9 h; 2-h PG_OGTT@fasting_, 2-hour plasma glucose during OGTT which was conducted in the fasting period (fasting time, ≥ 8 h); CI, confidence interval; HR, hazard ratio; OGTT, oral glucose tolerance test.

Model 1 was not adjusted; Model 2 was adjusted for age, sex, and ethnicity; Model 3 was adjusted for all the factors in Model 2 plus body mass index, poverty–income ratio, and education; Model 4 was adjusted for all the factors in Model 3 plus physical activity, alcohol consumption, smoking status, and survey period; Model 5 was adjusted for all the factors in Model 4 plus total cholesterol, HDL cholesterol, and systolic blood pressure; and Model 6 was adjusted for all the factors in Model 5 plus family history of diabetes.

**Table 5:** Association of 2-h plasma glucose during OGTT (square root-transformed) with CVD mortality.

Models	2-h PG_OGTT@4–7.9 h_			2-h PG_OGTT@fasting_		
	HR	95% CI	*p*	HR	95% CI	*p*
Model 1	1.13	1.10–1.17	< 0.001	1.12	1.10–1.15	< 0.001
Model 2	1.08	1.05–1.12	< 0.001	1.07	1.05–1.10	< 0.001
Model 3	1.07	1.04–1.11	< 0.001	1.07	1.04–1.10	< 0.001
Model 4	1.08	1.05–1.12	< 0.001	1.08	1.05–1.10	< 0.001
Model 5	1.07	1.03–1.11	< 0.001	1.06	1.03–1.09	< 0.001
Model 6	1.07	1.03–1.11	< 0.001	1.06	1.03–1.09	< 0.001

2-h PG_OGTT@4–7.9 h_, 2-hour plasma glucose during OGTT which was conducted in the postprandial period between 4 and 7.9 h; 2-h PG_OGTT@fasting_, 2-hour plasma glucose during OGTT which was conducted in the fasting period (fasting time, ≥ 8 h); CI, confidence interval; CVD, cardiovascular disease; HR, hazard ratio; OGTT, oral glucose tolerance test.

Model 1 was not adjusted; Model 2 was adjusted for age, sex, and ethnicity; Model 3 was adjusted for all the factors in Model 2 plus body mass index, poverty–income ratio, and education; Model 4 was adjusted for all the factors in Model 3 plus physical activity, alcohol consumption, smoking status, and survey period; Model 5 was adjusted for all the factors in Model 4 plus total cholesterol, HDL cholesterol, and systolic blood pressure; and Model 6 was adjusted for all the factors in Model 5 plus family history of diabetes.

Further analyses were conducted to characterize the association between 2-h plasma glucose during OGTT with mortality. 2-h PG_OGTT@4–7.9 h_ and 2-h PG_OGTT@fasting_ remained positively associated with higher risk for all-cause mortality and diabetes mortality after further adjustment for HbA_1c_ (Table 6). However, the positive association between 2-h plasma glucose and CVD mortality disappeared after this adjustment (Table 6). In addition, ROC curve analysis showed that the optimal cutoffs were 203.5 and 139.9 mg/dL, respectively, for 2-h PG_OGTT@4–7.9 h_ to classify diabetes mortality and CVD mortality (Supplementary Figures S3 & S4 online). Moreover, further analyses found that fasting status per se (4–7.9 h vs. ≥ 8 h) was not associated with mortality risks (Supplementary Table S9 online).

**Table 6:** Further analysis of the association between 2-h plasma glucose (square root-transformed) with mortality, with additional adjustment for HbA_1c_.

Models	2-h PG_OGTT@4–7.9 h_			2-h PG_OGTT@fasting_		
	HR	95% CI	*p*	HR	95% CI	*p*
All-cause mortality						
Full Model	1.06	1.04–1.08	< 0.001	1.06	1.04–1.07	< 0.001
Full Model + HbA_1c_	1.04	1.02–1.07	0.003	1.04	1.02–1.06	< 0.001
Diabetes mortality						
Full Model	1.46	1.33–1.61	< 0.001	1.29	1.21–1.38	< 0.001
Full Model + HbA_1c_	1.39	1.17–1.65	< 0.001	1.26	1.12–1.43	< 0.001
CVD mortality						
Full Model	1.07	1.03–1.11	< 0.001	1.06	1.03–1.09	< 0.001
Full Model + HbA_1c_	1.03	0.98–1.08	0.26	1.01	0.97–1.04	0.77
Cancer mortality						
Full Model	1.00	0.95–1.05	1.00	1.00	0.97–1.04	0.80
Full Model + HbA_1c_	1.02	0.96–1.07	0.58	1.00	0.96–1.05	0.85

2-h PG_OGTT@4–7.9 h_, 2-hour plasma glucose during OGTT which was conducted in the postprandial period between 4 and 7.9 h; 2-h PG_OGTT@fasting_, 2-hour plasma glucose during OGTT which was conducted in the fasting period (fasting time, ≥ 8 h); CI, confidence interval; HbA_1c_, hemoglobin A_1c_; HR, hazard ratio; OGTT, oral glucose tolerance test.

Full Model was adjusted for age, sex, ethnicity, body mass index, poverty–income ratio, education, physical activity, alcohol consumption, smoking status, survey period, total cholesterol, HDL cholesterol, systolic blood pressure, and family history of diabetes.

### Power and sample size estimation for 2-h PG_OGTT@4–7.9 h_ to diagnose diabetes

The current study has a limitation in evaluating the diagnostic utility of 2-h PG_OGTT@4–7.9 h_ within the postprandial cohort. In this analysis, diabetes was defined based on HbA_1c_ ≥ 6.5% or self-reported diagnosis, as fasting plasma glucose and standard fasting OGTT data were not available for this cohort. To rigorously assess the diagnostic utility of 2-h PG_OGTT@4–7.9 h_, future studies should be designed to incorporate all standard diabetes diagnostic parameters alongside the 2-h PG_OGTT@4–7.9 h_ measurement. Sample size estimation will be essential to ensure sufficient statistical power for these investigations.

Power analysis for using 2-h PG_OGTT@4–7.9 h_ to diagnose diabetes was conducted through the simulation of 10,000 random samples, and each simulation had a certain sample size ranging from 50 to 200 participants.

A diagnostic accuracy from 80% to 90% is considered excellent^12^. This study employed an accuracy threshold of 80% to conduct power and sample size estimations. Additionally, a slightly improved accuracy of 81% was also explored for these estimations (Supplementary Table S10). The findings suggested that a sample size of 100 participants may be necessary to achieve over 80% power in detecting a diagnostic accuracy of 81% using 2-h PG_OGTT@4–7.9 h_ in future studies (Supplementary Table S10).

## Discussion

Using a cohort of US adults (*n* = 2347), this study demonstrated, for the first time, that OGTT conducted during the postprandial period between 4 and 7.9 h may serve as a valuable tool for diabetes diagnosis and predicting mortality risk. This study suggests that non-fasting OGTT, which is more convenient than fasting OGTT, could be clinically significant.

2-h PG_OGTT@4–7.9 h_ classified HbA_1c_-diagnosed diabetes with 92% accuracy (95% CI 89%–94%), falling within the outstanding accuracy range (> 90%)^12^. This accuracy was comparable to its fasting counterpart, 2-h PG_OGTT@fasting_, which achieved 95% accuracy (95% CI, 93%–96%). We further conducted analysis using self-reported diagnosis of diabetes. In epidemiological studies, self-reported diagnosis of diabetes is widely accepted due to its relatively higher accuracy compared to many other chronic conditions such as stroke, heart disease, and hypertension^43,44^. Studies across diverse populations have consistently shown that self-reported diagnosis of diabetes exhibits a sensitivity of approximately 70%–75% in identifying true diabetes, with specificity exceeding 95%^45–48^. Our further analysis using self-reported diagnosis confirmed similar diagnostic accuracies between 2-h PG_OGTT@4–7.9 h_ and 2-h PG_OGTT@fasting_ (89% versus 88%). Therefore, our findings suggest that 2-h PG_OGTT@4–7.9 h_ holds promise as a diagnostic marker for diabetes.

The optimal cutoff for predicting HbA_1c_-diagnosed diabetes with 2-h PG_OGTT@4–7.9 h_ was 206.8 mg/dL, aligning closely with the cutoff for 2-h PG_OGTT@fasting_ at 203.6 mg/dL. This suggests that the clinical cutoff of 200 mg/dL used for 2-h PG_OGTT@fasting_^9,17,18^ may be applicable to 2-h PG_OGTT@4–7.9 h_ as well. Participants with 2-h PG_OGTT@4–7.9 h_ ≥ 200 mg/dL demonstrated a significantly higher risk of diabetes mortality (HR, 12.3; 95% CI, 5.4–27.9) compared to those with lower values (< 200 mg/dL).

2-h PG_OGTT@fasting_ is used to identify those at risk of prediabetes^49^. Here, we investigated whether 2-h PG_OGTT@4–7.9 h_ was associated with prediabetes, which was defined as HbA_1c_ ranging between 5.7% and 6.4%. We found that, in participants without diabetes (HbA_1c_ < 6.5%), those with 2-h PG_OGTT@4–7.9 h_ ≥140 mg/dL had a 29% higher adjusted risk of pre-diabetes compared with those with 2-h PG_OGTT@4–7.9 h_ <140 mg/dL. Therefore, 2-h PG_OGTT@4–7.9 h_ may be useful to identify those with a higher risk of pre-diabetes.

Regarding mortality predictions, both 2-h PG_OGTT@4–7.9 h_ and 2-h PG_OGTT@fasting_ effectively forecasted mortality from CVD and all causes. This is consistent with literature suggesting that 2-h PG_OGTT@fasting_ serves as an independent predictor for CVD^50–53^ and all-cause mortality^54–57^. Furthermore, 2-h PG_OGTT@4–7.9 h_ also demonstrated comparable predictive ability for mortality from CVD and all causes. It is important to highlight that the glycemic thresholds used for diabetes diagnosis are higher than those associated with elevated mortality risk. This suggests that current diagnostic criteria may be insufficient to fully capture cardiometabolic risk^58^. Moreover, given that individuals spend the majority of their time in a postprandial state—typically consuming three meals per day—there may be clinical relevance in considering postprandial glycemic measures for risk prediction.

Interestingly, neither 2-h PG_OGTT@4–7.9 h_ nor 2-h PG_OGTT@fasting_ predicted cancer mortality in this study, consistent with some reports in the literature regarding 2-h PG_OGTT@fasting_^59–61^. Notably, other studies have reported associations between 2-h PG_OGTT@fasting_ and cancer mortality^62,63^.

Moreover, both 2-h PG_OGTT@4–7.9 h_ and 2-h PG_OGTT@fasting_ predicted mortality specifically from diabetes, consistent with a previous report that 2-h PG_OGTT@fasting_ predicted diabetes mortality^64^. In fact, 2-h PG_OGTT@4–7.9 h_ exhibited potentially greater sensitivity for predicting diabetes mortality compared to its fasting counterpart, evidenced by an adjusted HR of 21.1 (95% CI, 9.2–48.0) versus 7.1 (95% CI, 4.2–11.9) per 1-square-root increase. A similar trend was observed when analyzing 2-h plasma glucose as a categorical variable (≥ versus < 200 mg/dL), with adjusted HRs of 12.3 (95% CI, 5.4–27.9) and 5.9 (95% CI, 3.4–10.1) for higher PG_OGTT@4–7.9 h_ and PG_OGTT@fasting_, respectively.

Many guidelines have started to recommend non-fasting lipids (total cholesterol, HDL & LDL cholesterol, and triglyceride) as the standard for cardiovascular risk assessment^10,65–67^. This is because non-fasting lipid tests are more comfortable and convenient for individuals than fasting tests. Importantly, non-fasting tests seem to have similar or better prognostic value for general risk screening^65,68^, CVD risks^10^ and all-cause mortality^68^ compared with their fasting counterpart.

The current study supports the shift from fasting to nonfasting OGTT tests, as 2-h PG_OGTT@4–7.9 h_ has a similar capacity in classifying diabetes diagnosis and in predicting mortality risks from CVD and all causes. The baseline glucose levels (mean ± standard deviation) prior to OGTT were 106.5 ± 34.3 mg/dL in the fasting cohort and 99.9 ± 32.2 mg/dL in the postprandial cohort. Although the 6.6 mg/dL difference was relatively small, it reached statistical significance (*p* < 0.05). Whether glucose levels during a fasting period of 4 to 7.9 h influence the accuracy of calculated insulin sensitivity and secretion indices remains to be determined. Further research is needed to assess the potential impact of fasting duration on these metabolic parameters.

In conclusion, this study found that 2-h PG_OGTT@4–7.9 h_ classified HbA_1c_-diagnosed diabetes with an outstanding accuracy of 92%, similar to that of 2-h PG_OGTT@fasting_ (i.e., 95%). 2-h PG_OGTT@4–7.9 h_ predicted mortality risk from diabetes, CVD and all causes. Therefore, 2-h PG_OGTT@4–7.9 h_, a non-fasting test, might be useful for diabetes classification and CVD risk prediction.

## Supplementary Information

Below is the link to the electronic supplementary material.

Supplementary Material 1

## Data Availability

All data in the current analysis are publicly available on the NHANES website.
