# Supplementary material for: Postprandial 2-h glucose tolerance is associated with diabetes diagnosis, diabetes mortality, and cardiovascular mortality
Source: Sci Rep. 2025 Dec 15;15:43853. doi: 10.1038/s41598-025-28849-y (PMC12705769; doi:10.1038/s41598-025-28849-y)
Supplement: Supplementary file 1 — Supplementary Material 1 [file 41598_2025_28849_MOESM1_ESM.docx]

Supplementary Materials

**Postprandial two-hour glucose tolerance is associated with diabetes diagnosis, diabetes mortality, and cardiovascular mortality**

Authors: Yutang Wang, Yan Fang, Guang Yang, Francesco Prattichizzo, **Antonio Ceriello**


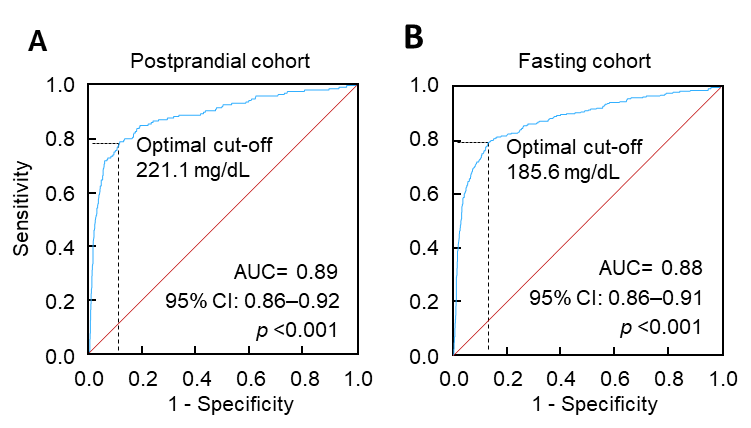


**Supplementary Figure S1.** ROC curves of 2-h plasma glucose to classify diabetes, defined as a self-reported diagnosis. **A**, OGTT was conducted in the postprandial period between 4 and 7.9 h. The optimal cutoff was 221.1 mg/dL, with a sensitivity of 79.0%, specificity of 88.7%, and an area under the curve (AUC) of 0.89. **B**, OGTT was conducted in the fasting period (fasting time, ≥ 8 h). The optimal cutoff was 185.6 mg/dL, with a sensitivity of 79.5%, specificity of 87.0%, and an AUC of 0.88. OGTT, oral glucose tolerance test; ROC, receiver operating characteristic.


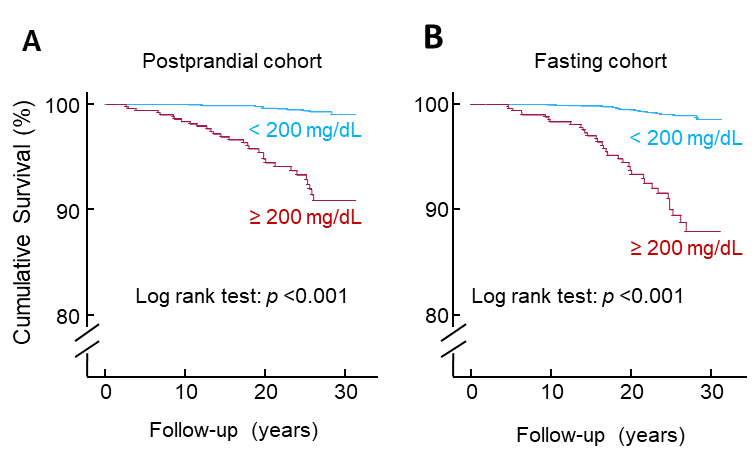


**Supplementary Figure S2**. Kaplan–Meier survival curves of 2-h plasma glucose for diabetes mortality. **A**, The postprandial cohort; **B**, The fasting cohort. The 2-h plasma glucose during OGTT was stratified as ≥ versus < 200 mg/dL. OGTT, oral glucose tolerance test.


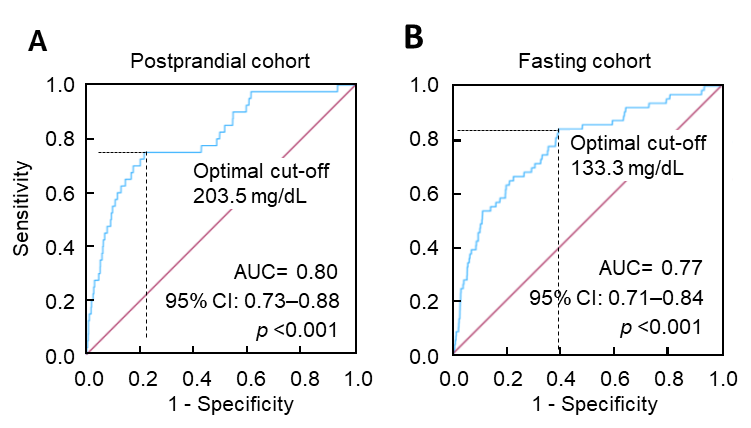


**Supplementary Figure S3.** ROC curves of 2-h plasma glucose to classify diabetes mortality. **A**, OGTT was conducted in the postprandial period between 4 and 7.9 h. The optimal cutoff was 203.5 mg/dL, with a sensitivity of 75.0%, specificity of 78.6%, and an area under the curve (AUC) of 0.80. **B**, OGTT was conducted in the fasting period (fasting time, ≥ 8 h). The optimal cutoff was 133.3 mg/dL, with a sensitivity of 83.9%, specificity of 60.6%, and an AUC of 0.77. OGTT, oral glucose tolerance test; ROC, receiver operating characteristic.

**
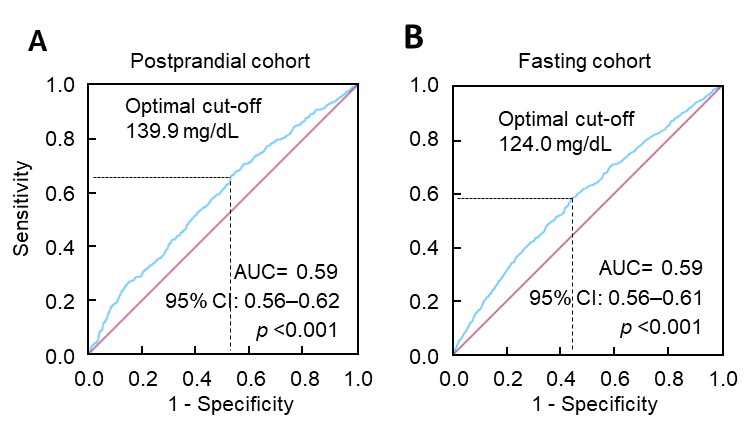
**

**Supplementary Figure S4.** ROC curves of 2-h plasma glucose to classify cardiovascular mortality. **A**, OGTT was conducted in the postprandial period between 4 and 7.9 h. The optimal cutoff was 139.9 mg/dL, with a sensitivity of 66.0%, specificity of 47.4%, and an area under the curve (AUC) of 0.59. **B**, OGTT was conducted in the fasting period (fasting time, ≥ 8 h). The optimal cutoff was 124.0 mg/dL, with a sensitivity of 58.3%, specificity of 55.9%, and an AUC of 0.59. OGTT, oral glucose tolerance test; ROC, receiver operating characteristic.

**Supplementary Table S1.** Baseline characteristics of the postprandial cohort (fasting time, 4–7.9 h).

|  | 2-h PG_OGTT@4–7.9h_ | | All | *p* |
| --- | --- | --- | --- | --- |
|  | < 200 mg/dL | ≥ 200 mg/dL |  |  |
| Sample size | 1797 | 550 | 2347 | NA |
| Age, y, mean (SD) | 55 (11) | 61 (9) | 56 (11) | <0.001 |
| Sex (male), *n* (%) | 869 (48.4) | 246 (44.7) | 1115 (47.5) | 0.14 |
| 2-h PG_OGTT@4–7.9h_, mg/dL, median (IQR) | 131 (105–158) | 247 (218–303) | 147 (114–196) | <0.001 |
| HbA_1c_, %, median (IQR) | 5.4 (5.1–5.7) | 6.0 (5.4–7.1)) | 5.5 (5.1–5.8) | <0.001 |
| BMI, kg/m^2^, median (IQR) | 27 (24–30) | 28 (25–32) | 27 (24–31) | <0.001 |
| TC, mg/dL, median (IQR) | 212 (185–242) | 226 (199–253) | 215 (189–245) | <0.001 |
| HDL-C, mg/dL, median (IQR) | 49 (40–60) | 47 (38–57) | 49 (40–59) | 0.003 |
| SBP, mm Hg, median (IQR) | 127 (117–140) | 136 (126–151) | 129 (118–142) | <0.001 |
| Ethnicity, *n* (%) |  |  |  |  |
| Non-Hispanic white | 928 (51.6) | 245 (44.5) | 1173 (50) | <0.001 |
| Non-Hispanic black | 408 (22.7) | 100 (18.2) | 508 (21.6) |  |
| Hispanic | 436 (24.3) | 198 (36.0) | 634 (27.0) |  |
| Other | 25 (1.4) | 7 (1.3) | 32 (1.4) |  |
| Education, *n* (%) |  |  |  |  |
| <High school | 674 (37.5) | 276 (50.2) | 950 (40.5) | <0.001 |
| High school | 551 (30.7) | 151 (27.5) | 702 (29.9) |  |
| >High school | 561 (31.2) | 120 (21.8) | 681 (29.0) |  |
| Unknown | 11 (0.6) | 3 (0.5) | 14 (0.6) |  |
| Poverty–income ratio, *n* (%) |  |  |  |  |
| <130% | 372 (20.7) | 139 (25.3) | 511 (21.8) | 0.002 |
| 130%–349% | 746 (41.5) | 236 (42.9) | 982 (41.8) |  |
| ≥350% | 553 (30.8) | 127 (23.1) | 680 (29.0) |  |
| Unknown | 126 (7.0) | 48 (8.7) | 174 (7.4) |  |
| Physical activity, *n* (%) |  |  |  |  |
| Active | 667 (37.1) | 192 (34.9) | 859 (36.6) | 0.56 |
| Insufficiently active | 796 (44.3) | 247 (44.9) | 1043 (44.4) |  |
| Inactive | 334 (18.6) | 111 (20.2) | 445 (19.0) |  |
| Alcohol consumption, *n* (%) |  |  |  |  |
| 0 drinks/week | 277 (15.4) | 121 (22.0) | 398 (17.0) | <0.001 |
| <1 drink/week | 228 (12.7) | 53 (9.6) | 281 (12.0) |  |
| 1–6 drinks/week | 345 (19.2) | 71 (12.9) | 416 (17.7) |  |
| ≥7 drinks/week | 222 (12.4) | 62 (11.3) | 284 (12.1) |  |
| Unknown | 725 (40.3) | 243 (44.2) | 968 (41.2) |  |
| Smoking status, *n* (%) |  |  |  |  |
| Current smoker | 459 (25.5) | 78 (14.2) | 537 (22.9) | <0.001 |
| Past smoker | 547 (30.4) | 209 (38.0) | 756 (32.2) |  |
| Non-smoker | 791 (44.0) | 263 (47.8) | 1054 (44.9) |  |
| Survey period, *n* (%) |  |  |  |  |
| 1988–1991 | 873 (48.6) | 253 (46.0) | 1126 (48.0) | 0.29 |
| 1991–1994 | 924 (51.4) | 297 (54.0) | 1221 (52.0) |  |
| Family history of diabetes, *n* (%) |  |  |  |  |
| Yes | 787 (43.8) | 290 (52.7) | 1077 (45.9) | <0.001 |
| No | 990 (55.1) | 249 (45.3) | 1239 (52.8) |  |
| Unknown | 20 (1.1) | 11 (2.0) | 31 (1.3) |  |
| Selt reported diagnosis, *n* (%) |  |  |  |  |
| Yes | 29 (1.6) | 152 (27.6) | 181 (7.7) | <0.001 |
| No | 1767 (98.3) | 398 (72.4) | 2165 (92.2) |  |
| Unknown | 1 (0.1) | 0 (0.0) | 1 (0.0) |  |

Abbreviations: 2-h PG_OGTT@4–7.9h_, 2-hour plasma glucose during OGTT which was conducted in the postprandial period between 4 and 7.9 h; BMI, body mass index; HbA_1c_, hemoglobin A_1c_; HDL-C, high-density lipoprotein cholesterol; IQR, interquartile range; NA, not applicable; OGTT, oral glucose tolerance test; SBP, systolic blood pressure; SD, standard deviation; TC, total cholesterol.

**Supplementary Table S2.** Baseline characteristics of the fasting cohort (fasting time, ≥8 h)

|  | 2-h PG_OGTT@fasting_ | | All | *p* |
| --- | --- | --- | --- | --- |
|  | < 200 mg/dL | ≥ 200 mg/dL |  |  |
| Sample size | 3287 | 578 | 3865 | NA |
| Age, y, mean (SD) | 55 (10) | 60 (9) | 56 (10) | <0.001 |
| Sex (male), *n* (%) | 1614 (49.1) | 292 (50.5) | 1906 (49.3) | 0.53 |
| 2-h PG_OGTT@fasting_, mg/dL, median (IQR) | 114 (93–140) | 275 (224–353) | 121 (97–162) | <0.001 |
| HbA_1c_, %, median (IQR) | 5.4 (5.1–5.7) | 6.7 (5.9–8.4) | 5.5 (5.2–5.9) | <0.001 |
| BMI, kg/m^2^, median (IQR) | 27 (24–31) | 30 (26–33) | 27 (24–31) | <0.001 |
| TC, mg/dL, median (IQR) | 213 (188–240) | 222 (194–251) | 214 (189–242) | <0.001 |
| HDL-C, mg/dL, median (IQR) | 49 (40–60) | 44 (36–54) | 48 (39–59) | <0.001 |
| SBP, mm Hg, median (IQR) | 126 (115–139) | 136 (126–150) | 128 (117–141) | <0.001 |
| Ethnicity, *n* (%) |  |  |  |  |
| Non-Hispanic white | 1527 (46.5) | 216 (37.4) | 1743 (45.1) | <0.001 |
| Non-Hispanic black | 864 (26.3) | 126 (21.8) | 990 (25.6) |  |
| Hispanic | 847 (25.8) | 231 (40.0) | 1078 (27.9) |  |
| Other | 49 (1.5) | 5 (0.9) | 54 (1.4) |  |
| Education, *n* (%) |  |  |  |  |
| <High school | 1350 (41.1) | 332 (57.4) | 1682 (43.5) | <0.001 |
| High school | 977 (29.7) | 150 (26.0) | 1127 (29.2) |  |
| >High school | 939 (28.6) | 96 (16.6) | 1035 (26.8) |  |
| Unknown | 21 (0.6) | 0 (0) | 21 (0.5) |  |
| Poverty–income ratio, *n* (%) |  |  |  |  |
| <130% | 766 (23.3) | 197 (34.1) | 963 (24.9) | <0.001 |
| 130%–349% | 1308 (39.8) | 218 (37.7) | 1526 (39.5) |  |
| ≥350% | 902 (27.4) | 101 (17.5) | 1003 (26.0) |  |
| Unknown | 311 (9.5) | 62 (10.7) | 373 (9.7) |  |
| Physical activity, *n* (%) |  |  |  |  |
| Active | 1198 (36.4) | 187 (32.4) | 1385 (35.8) | 0.08 |
| Insufficiently active | 1397 (42.5) | 249 (43.1) | 1646 (42.6) |  |
| Inactive | 692 (21.1) | 142 (24.6) | 834 (21.6) |  |
| Alcohol consumption, *n* (%) |  |  |  |  |
| 0 drinks/week | 467 (14.2) | 118 (20.4) | 585 (15.1) | <0.001 |
| <1 drink/week | 391 (11.9) | 50 (8.7) | 441 (11.4) |  |
| 1–6 drinks/week | 612 (18.6) | 76 (13.1) | 688 (17.8) |  |
| ≥7 drinks/week | 409 (12.4) | 66 (11.4) | 475 (12.3) |  |
| Unknown | 1408 (42.8) | 268 (46.4) | 1676 (43.4) |  |
| Smoking status, *n* (%) |  |  |  |  |
| Current smoker | 910 (27.7) | 112 (19.4) | 1022 (26.4) | <0.001 |
| Past smoker | 1015 (30.9) | 231 (40.0) | 1246 (32.2) |  |
| Non-smoker | 1362 (41.4) | 235 (40.7) | 1597 (41.3) |  |
| Survey period, *n* (%) |  |  |  |  |
| 1988–1991 | 1584 (48.2) | 261 (45.2) | 1845 (47.7) | 0.18 |
| 1991–1994 | 1703 (51.8) | 317 (54.8) | 2020 (52.3) |  |
| Family history of diabetes, *n* (%) |  |  |  |  |
| Yes | 1435 (43.7) | 309 (53.5) | 1744 (45.1) | <0.001 |
| No | 1814 (55.2) | 262 (45.3) | 2076 (53.7) |  |
| Unknown | 38 (1.2) | 7 (1.2) | 45 (1.2) |  |
| Selt reported diagnosis, *n* (%) |  |  |  |  |
| Yes | 76 (2.3) | 212 (36.7) | 288 (7.5) | <0.001 |
| No | 3207 (97.6) | 365 (63.1) | 3572 (92.4) |  |
| Unknown | 4 (0.1) | 1 (0.2) | 5 (0.1) |  |

Abbreviations: 2-h PG_OGTT@fasting_, 2-hour plasma glucose during OGTT which was conducted in the fasting period (fasting time, ≥ 8 h); BMI, body mass index; HbA_1c_, hemoglobin A_1c_; HDL-C, high-density lipoprotein cholesterol; IQR, interquartile range; NA, not applicable; OGTT, oral glucose tolerance test; SBP, systolic blood pressure; SD, standard deviation; TC, total cholesterol.

**Supplementary Table S3.** Association of 2-h plasma glucose during OGTT^1^ (independent variable) with HbA_1c_^2^ (dependent variable)

| Models | 2-h PG_OGTT@4–7.9h_ | | 2-h PG_OGTT@fasting_ | |
| --- | --- | --- | --- | --- |
|  | β | *p* | β | *p* |
| Model 1 | 0.614 | <0.001 | 0.671 | <0.001 |
| Model 2 | 0.616 | <0.001 | 0.661 | <0.001 |
| Model 3 | 0.603 | <0.001 | 0.651 | <0.001 |
| Model 4 | 0.609 | <0.001 | 0.656 | <0.001 |
| Model 5 | 0.608 | <0.001 | 0.653 | <0.001 |
| Model 6 | 0.606 | <0.001 | 0.650 | <0.001 |

2-h PG_OGTT@4–7.9h_, 2-hour plasma glucose during OGTT which was conducted in the postprandial period between 4 and 7.9 h; 2-h PG_OGTT@fasting_, 2-hour plasma glucose during OGTT which was conducted in the fasting period (fasting time, ≥ 8 h); HbA_1c_, hemoglobin A_1c_; OGTT, oral glucose tolerance test.

^1^ square root-transformed.

^2^ Natural log-transformed.

Model 1 was not adjusted; Model 2 was adjusted for age, sex, and ethnicity; Model 3 was adjusted for all the factors in Model 2 plus body mass index, poverty–income ratio, and education; Model 4 was adjusted for all the factors in Model 3 plus physical activity, alcohol consumption, smoking status, and survey period; Model 5 was adjusted for all the factors in Model 4 plus total cholesterol, HDL cholesterol, and systolic blood pressure; and Model 6 was adjusted for all the factors in Model 5 plus family history of diabetes.

**Supplementary Table S4.** Association of 2-h plasma glucose (square root-transformed) with diabetes (defined as a self-reported diagnosis).

| Models | 2-h PG_OGTT@4–7.9h_ (*n* = 2346^1^) | | | 2-h PG_OGTT@fasting_ (*n* = 3860^2^) | | |
| --- | --- | --- | --- | --- | --- | --- |
|  | OR | 95% CI | *p* | OR | 95% CI | *p* |
| Model 1 | 1.84 | 1.71–1.97 | <0.001 | 1.63 | 1.56–1.70 | <0.001 |
| Model 2 | 1.83 | 1.70–1.96 | <0.001 | 1.61 | 1.54–1.68 | <0.001 |
| Model 3 | 1.82 | 1.69–1.96 | <0.001 | 1.6 | 1.53–1.67 | <0.001 |
| Model 4 | 1.84 | 1.70–1.99 | <0.001 | 1.6 | 1.53–1.67 | <0.001 |
| Model 5 | 1.86 | 1.72–2.02 | <0.001 | 1.6 | 1.53–1.67 | <0.001 |
| Model 6 | 1.86 | 1.71–2.02 | <0.001 | 1.58 | 1.51–1.66 | <0.001 |

2-h PG_OGTT@4–7.9h_, 2-hour plasma glucose during OGTT which was conducted in the postprandial period between 4 and 7.9 h; 2-h PG_OGTT@fasting_, 2-hour plasma glucose during OGTT which was conducted in the fasting period (fasting time, ≥ 8 h); CI, confidence interval; OGTT, oral glucose tolerance test; OR, odds ratio.

^1^ Out of 2347 participants, the self-reported diabetes status was missing in one participant. Therefore, the remaining 2346 participants were included in the analysis.

^2^ Out of 3865 participants, the self-reported diabetes status was missing in 5 participants. Therefore, the remaining 3860 participants were included in the analysis.

Model 1 was not adjusted; Model 2 was adjusted for age, sex, and ethnicity; Model 3 was adjusted for all the factors in Model 2 plus body mass index, poverty–income ratio, and education; Model 4 was adjusted for all the factors in Model 3 plus physical activity, alcohol consumption, smoking status, and survey period; Model 5 was adjusted for all the factors in Model 4 plus total cholesterol, HDL cholesterol, and systolic blood pressure; and Model 6 was adjusted for all the factors in Model 5 plus family history of diabetes.

**Supplementary Table S5.** Association of plasma glucose during OGTT (square root-transformed) with pre-diabetes diagnosis

| Models | 2-h PG_OGTT@4–7.9h_ | | | 2-h PG_OGTT@fasting_ | | |
| --- | --- | --- | --- | --- | --- | --- |
|  | OR | 95% CI | *p* | OR | 95% CI | *p* |
| Model 1 | 1.15 | 1.10–1.21 | <0.001 | 1.25 | 1.21-1.30 | <.001 |
| Model 2 | 1.15 | 1.09–1.21 | <0.001 | 1.22 | 1.17-1.27 | <.001 |
| Model 3 | 1.13 | 1.07–1.19 | <0.001 | 1.20 | 1.15-1.25 | <.001 |
| Model 4 | 1.15 | 1.09–1.21 | <0.001 | 1.22 | 1.17-1.27 | <.001 |
| Model 5 | 1.13 | 1.07–1.19 | <0.001 | 1.22 | 1.17-1.27 | <.001 |
| Model 6 | 1.13 | 1.07–1.19 | <0.001 | 1.22 | 1.16-1.27 | <.001 |

Pre-diabetes diagnosis was defined as HbA_1c_ ranging between 5.7% and 6.4%. Out of 2347 participants in the postprandial cohort, 230 had an HbA_1c_ value of ≥6.5% and were excluded from the analysis. So, a total of 2117 participants were included in the final analysis. 2-h PG_OGTT@4–7.9h_, 2-hour plasma glucose during OGTT which was conducted in the postprandial period between 4 and 7.9 h; 2-h PG_OGTT@fasting_, 2-hour plasma glucose during OGTT which was conducted in the fasting period (fasting time, ≥ 8 h); CI, confidence interval; HbA_1c_, hemoglobin A_1c_; OGTT, oral glucose tolerance test; OR, odds ratio.

Model 1 was not adjusted; Model 2 was adjusted for age, sex, and ethnicity; Model 3 was adjusted for all the factors in Model 2 plus body mass index, poverty–income ratio, and education; Model 4 was adjusted for all the factors in Model 3 plus physical activity, alcohol consumption, smoking status, and survey period; Model 5 was adjusted for all the factors in Model 4 plus total cholesterol, HDL cholesterol, and systolic blood pressure; and Model 6 was adjusted for all the factors in Model 5 plus family history of diabetes.

**Supplementary Table S6**. Numbers of mortality during the follow-up

| Mortality | Postprandial cohort | Fasting cohort | All |
| --- | --- | --- | --- |
| All causes | 1299 | 2144 | 3443 |
| Diabetes | 40 | 62 | 102 |
| CVD | 432 | 734 | 1166 |
| Cancer | 319 | 562 | 881 |

CVD, cardiovascular disease.

**Supplementary Table S7.** Association of 2-h plasma glucose during OGTT (≥ versus < 200 mg/dL) with diabetes mortality.

| Models | 2-h PG_OGTT@4–7.9h_ | | | 2-h PG_OGTT@fasting_ | | |
| --- | --- | --- | --- | --- | --- | --- |
|  | HR | 95% CI | *p* | HR | 95% CI | *p* |
| Model 1 | 12.4 | 6.0–25.4 | <0.001 | 10.4 | 6.3–17.2 | <0.001 |
| Model 2 | 10.0 | 4.7–20.9 | <0.001 | 8.1 | 4.8–13.7 | <0.001 |
| Model 3 | 9.0 | 4.3–19.1 | <0.001 | 7.3 | 4.3–12.4 | <0.001 |
| Model 4 | 13.7 | 6.2–30.6 | <0.001 | 7.3 | 4.3–12.5 | <0.001 |
| Model 5 | 12.0 | 5.3–27.1 | <0.001 | 6.1 | 3.6–10.4 | <0.001 |
| Model 6 | 12.3 | 5.4–27.9 | <0.001 | 5.9 | 3.4–10.1 | <0.001 |

2-h PG_OGTT@4–7.9h_, 2-hour plasma glucose during OGTT which was conducted in the postprandial period between 4 and 7.9 h; 2-h PG_OGTT@fasting_, 2-hour plasma glucose during OGTT which was conducted in the fasting period (fasting time, ≥ 8 h); CI, confidence interval; HR, hazard ratio; OGTT, oral glucose tolerance test.

Model 1 was not adjusted; Model 2 was adjusted for age, sex, and ethnicity; Model 3 was adjusted for all the factors in Model 2 plus body mass index, poverty–income ratio, and education; Model 4 was adjusted for all the factors in Model 3 plus physical activity, alcohol consumption, smoking status, and survey period; Model 5 was adjusted for all the factors in Model 4 plus total cholesterol, HDL cholesterol, and systolic blood pressure; and Model 6 was adjusted for all the factors in Model 5 plus family history of diabetes.

**Supplementary Table S8.** Association of 2-h plasma glucose during OGTT (square root-transformed) with cancer mortality.

| Models | 2-h PG_OGTT@4–7.9h_ | | | 2-h PG_OGTT@fasting_ | | |
| --- | --- | --- | --- | --- | --- | --- |
|  | HR | 95% CI | *p* | HR | 95% CI | *p* |
| Model 1 | 1.02 | 0.97–1.06 | 0.47 | 1.04 | 1.01–1.07 | 0.02 |
| Model 2 | 0.98 | 0.94–1.03 | 0.37 | 1.00 | 0.97–1.03 | 0.96 |
| Model 3 | 0.99 | 0.94–1.03 | 0.53 | 1.00 | 0.97–1.04 | 0.89 |
| Model 4 | 1.00 | 0.96–1.05 | 0.90 | 1.02 | 0.98–1.05 | 0.38 |
| Model 5 | 1.00 | 0.95–1.04 | 0.88 | 1.01 | 0.98–1.04 | 0.62 |
| Model 6 | 1.00 | 0.95–1.05 | 1.00 | 1.00 | 0.97–1.04 | 0.80 |

2-h PG_OGTT@4–7.9h_, 2-hour plasma glucose during OGTT which was conducted in the postprandial period between 4 and 7.9 h; 2-h PG_OGTT@fasting_, 2-hour plasma glucose during OGTT which was conducted in the fasting period (fasting time, ≥ 8 h); CI, confidence interval; HR, hazard ratio; OGTT, oral glucose tolerance test.

Model 1 was not adjusted; Model 2 was adjusted for age, sex, and ethnicity; Model 3 was adjusted for all the factors in Model 2 plus body mass index, poverty–income ratio, and education; Model 4 was adjusted for all the factors in Model 3 plus physical activity, alcohol consumption, smoking status, and survey period; Model 5 was adjusted for all the factors in Model 4 plus total cholesterol, HDL cholesterol, and systolic blood pressure; and Model 6 was adjusted for all the factors in Model 5 plus family history of diabetes.

**Supplementary Table S9.** Association of fasting status (4–7.9 h vs ≥ 8 h) with mortality

| Mortality | Model 1 | | | Model 2 | | |
| --- | --- | --- | --- | --- | --- | --- |
|  | HR | 95% CI | *p* | HR | 95% CI | *p* |
| All-cause mortality |  |  |  |  |  |  |
| Reference (≥ 8h) | 1.00 |  |  | 1.00 |  |  |
| 4–7.9h | 0.98 | 0.92–1.05 | 0.62 | 0.94 | 0.87–1.01 | 0.07 |
| Diabetes mortality |  |  |  |  |  |  |
| Reference (≥ 8h) | 1.00 |  |  | 1.00 |  |  |
| 4–7.9h | 1.02 | 0.68–1.52 | 0.94 | 0.86 | 0.57–1.30 | 0.48 |
| CVD mortality |  |  |  |  |  |  |
| Reference (≥ 8h) | 1.00 |  |  | 1.00 |  |  |
| 4–7.9h | 0.94 | 0.83–1.06 | 0.30 | 0.89 | 0.79–1.01 | 0.07 |
| Cancer mortality |  |  |  |  |  |  |
| Reference (≥ 8h) | 1.00 |  |  | 1.00 |  |  |
| 4–7.9h | 1.07 | 0.81–1.40 | 0.65 | 0.95 | 0.83–1.10 | 0.50 |

CI, confidence interval; HR, hazard ratio; OGTT, oral glucose tolerance test.

Model 1 was adjusted for age, sex, ethnicity, body mass index, poverty–income ratio, education, physical activity, alcohol consumption, smoking status, survey period, total cholesterol, HDL cholesterol, systolic blood pressure, and family history of diabetes. Model 2 was adjusted for all the factors in Model 1 plus 2-h plasma glucose during OGTT (square root-transformed).

**Supplementary Table S10.** Power estimation for 2-h PG_OGTT@4–7.9h_ to diagnose diabetes^1^

| Sample size | *n* = 50 | *n* = 90 | *n* = 100 | *n* = 150 | *n* = 175 | *n* = 200 |
| --- | --- | --- | --- | --- | --- | --- |
| Power for  80% accuracy | 82.8% | 86.4% | 87.8% | 91.8% | 92.8% | 94.1% |
| Power for  81% accuracy | 71.2% | 79.3% | 81.7% | 83.1% | 85.8% | 88.0% |
| Sensitivity  (95% CI) | 86.5%  (50.0%–100%) | 86.5%  (60.0%–100%) | 86.6%  (61.5%–100%) | 86.5%  (66.7%–100%) | 86.5%  (68.8%–100%) | 86.6%  (70.0%–100%) |
| Specificity  (95% CI) | 83.5%  (72.1%–93.5%) | 83.4%  (75.3%–91.0%) | 83.4%  (75.8%–90.4%) | 83.4%  (77.3%–89.4%) | 83.4%  (77.6%–88.7%) | 83.4%  (78.0%–88.5%) |

2-h PG_OGTT@4–7.9h_, 2-hour plasma glucose during OGTT which was conducted in the postprandial period between 4 and 7.9 h; CI, confidence interval; OGTT, oral glucose tolerance test.

^1^ Diabetes was defined as HbA_1c_ ≥6.5%

Power was estimated using simulations on 10,000 random samples for each sample size.
